# FastD: Fast detection of insecticide target‐site mutations and overexpressed detoxification genes in insect populations from RNA‐Seq data

**DOI:** 10.1002/ece3.7037

**Published:** 2020-11-21

**Authors:** Longfei Chen, Kun Lang, Yang Mei, Zhenmin Shi, Kang He, Fei Li, Huamei Xiao, Gongyin Ye, Zhaojun Han

**Affiliations:** ^1^ Institute of Insect Sciences College of Agriculture and Biotechnology Zhejiang University Hangzhou China; ^2^ Department of Entomology Nanjing Agricultural University Nanjing China; ^3^ Key Laboratory of Crop Growth and Development Regulation of Jiangxi Province College of Life Sciences and Resource Environment Yichun University Yichun China

**Keywords:** detoxification genes, insecticide resistance, RNA‐Seq, target‐site mutations, webserver

## Abstract

Target‐site mutations and detoxification gene overexpression are two major mechanisms conferring insecticide resistance. Molecular assays applied to detect these resistance genetic markers are time‐consuming and with high false‐positive rates. RNA‐Seq data contains information on the variations within expressed genomic regions and expression of detoxification genes. However, there is no corresponding method to detect resistance markers at present. Here, we collected 66 reported resistance mutations of four insecticide targets (AChE, VGSC, RyR, and nAChR) from 82 insect species. Next, we obtained 403 sequences of the four target genes and 12,665 sequences of three kinds of detoxification genes including P450s, GSTs, and CCEs. Then, we developed a Perl program, FastD, to detect target‐site mutations and overexpressed detoxification genes from RNA‐Seq data and constructed a web server for FastD (http://www.insect-genome.com/fastd). The estimation of FastD on simulated RNA‐Seq data showed high sensitivity and specificity. We applied FastD to detect resistant markers in 15 populations of six insects, *Plutella xylostella*, *Aphis gossypii*, *Anopheles arabiensis*, *Musca domestica*, *Leptinotarsa decemlineata* and *Apis mellifera*. Results showed that 11 RyR mutations in *P. xylostella*, one nAChR mutation in *A. gossypii*, one VGSC mutation in *A. arabiensis* and five VGSC mutations in *M. domestica* were found to be with frequency difference >40% between resistant and susceptible populations including previously confirmed mutations G4946E in RyR, R81T in nAChR and L1014F in VGSC. And 49 detoxification genes were found to be overexpressed in resistant populations compared with susceptible populations including previously confirmed detoxification genes *CYP6BG1*, *CYP6CY22*, *CYP6CY13*, *CYP6P3*, *CYP6M2*, *CYP6P4* and *CYP4G16*. The candidate target‐site mutations and detoxification genes were worth further validation. Resistance estimates according to confirmed markers were consistent with population phenotypes, confirming the reliability of this program in predicting population resistance at omics‐level.

## Graphical abstract

We developed a program called FastD to detect the insecticide target‐site mutations and overexpressed detoxification genes from RNA‐Seq data and constructed a corresponding web server for FastD (http://www.insect-genome.com/fastd) in this article.

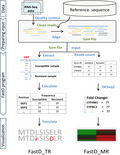

## 1 INTRODUCTION

Insect pests have a great impact on many aspects of human life. Among all of these aspects, the harm to human health and the yield loss in agricultural production are the most concerning. To make matters worse, some insects serve as medium of pathogens, spreading diseases and causing damage simultaneously. For example, *Anopheles gambiae* spread malaria and caused millions of deaths annually in Africa (Consortium, 2017). As for agricultural production, the estimated yield loss of crops due to insect pests is over 18% globally (Oerke, 2005).

Although there are many insect pest control methods available, application of insecticides is still one of the most frequently used methods. Chemical insecticides were first introduced to control insect pests in the 1940s. Since then, thousands of insecticides have been developed to protect human health and crops. Unfortunately, long‐term mismanagement of insecticide application led to the development of insecticide resistance within insect pest populations. So far, more than 553 insect species have been reported to have developed resistance to approximately 331 insecticides (Gould et al., 2018). The development of insecticide resistance necessitates the application of higher dosages of said insecticide for controlling insect pests, which in turn causes more serious threats to human and environmental health (Kim et al., 2017; Tang et al., 2018). Insecticide resistance has become one of the most formidable obstacles in insect pest control (Gould et al., 2018).

Insecticide target‐site mutations and overexpression of detoxification gene(s) are two major mechanisms conferring insecticide resistance (Ffrench‐Constant, 2013). Due to long‐term selection by insecticides, the individuals containing resistance‐associated genotypes rapidly accumulate within populations. Generally, insecticide resistance of insect pest populations can be predicted according to the prevalence of resistance markers including target‐site mutations and overexpressed detoxification genes (Sonoda, 2010). To date, most resistance cases occurred within five classes of insecticides: organophosphates, pyrethroids, carbamates, neonicotinoids and diamides (Thomas & Ralf, 2015). According to the modes of action listed by the Insecticide Resistance Action Committee (IRAC), organophosphates and carbamates target acetylcholinesterases (AChE), pyrethroids target voltage gated sodium channels (VGSC), diamides target ryanodine receptors (RyR) and neonicotinoids target nicotinic acetylcholine receptor (nAChR). In addition, metabolic resistance of these five classes of insecticides are mainly associated with three important detoxification gene families: cytochrome P450 (P450), glutathione S‐transferase (GST) and carboxyl/cholinesterases (CCE) (Yan et al., 2012).

Detecting target‐site mutations and overexpressed detoxification genes within insect pest populations has long been a useful method in monitoring resistance. Many methods have been developed to detect target mutations such as PCR amplification of specific alleles (PASA) (Yan et al., 2014) and random amplified polymorphic DNA (RAPD) (Ferguson & Pineda, 2010). DNA microarray has been used to detect overexpressed detoxification genes (Mavridis et al., 2019). However, these methods are inefficient and time‐consuming.

RNA‐Seq data contains information allowing detection of single‐nucleotide polymorphisms (SNPs) and gene expression levels (Costa et al., 2010). Thus, RNA‐Seq data can be used to detect resistance markers including target‐site mutations and overexpressed detoxification genes (Bonizzoni et al., 2015; De Wit et al., 2015). Here, to monitor the resistance of the aforementioned five classes of insecticides, we collected reported target‐site mutations, target gene allelic sequences and three groups of detoxification gene sequences from 82 insect species, and then developed a program, FastD, to detect target‐site mutations and overexpressed detoxification genes from RNA‐Seq data. To validate the reliability, we applied FastD to detect target‐site mutations and overexpressed detoxification genes in 15 populations of six insect species with both RNA‐Seq data and resistance phenotypes submitted to NCBI, including *Plutella xylostella*, *Aphis gossypii*, *Anopheles arabiensis*, *Musca domestica*, *Leptinotarsa decemlineata* and *Apis mellifera*.

## 2 MATERIALS AND METHODS

### 2.1 Resistance‐associated target‐site mutations collection by literature mining

To obtain the previously confirmed mutations in four targets associated with insecticide resistance, we first collected published literature from the NCBI PubMed database. For collection of literature relevant to target mutations in VGSC, we searched against NCBI PubMed with the term: ((“VGSC” [Abstract]) OR “voltage gated sodium channel” [Abstract]) AND “insecticide resistance” [Abstract]). For collection of literature relevant to target mutations in AChE, we searched against PubMed with the term: ((“AChE” [Abstract]) OR “acetylcholinesterase” [Abstract]) AND “insecticide resistance” [Abstract]). For collection of literature relevant to target mutations in RyR, we searched against PubMed with the term: ((“RyR” [Abstract]) OR “ryanodine receptor” [Abstract]) AND “insecticide resistance” [Abstract]). Finally, for collection of literature relevant to target mutations in nAChR, we searched against PubMed with the term: ((“nAChR” [Abstract]) OR “nicotinic acetylcholine receptor” [Abstract]) AND “insecticide resistance” [Abstract]).

### 2.2 Resistance‐associated gene sequences

We collected corresponding gene sequences from 82 insect species: 26 Hymenopterans, 21 Dipterans, 14 Lepidopterans, 10 Hemipterans, 6 Coleopterans, and 5 of other orders from NCBI and InsectBase (Yin et al., 2016). According to the two main mechanisms of insecticide resistance, resistance‐associated genes generally include two types: target genes and detoxification genes. To collect target gene sequences, we downloaded the confirmed full cDNA sequences of *VGSC*, *AChE*, *RyR*, and *nAChR* from InsectBase. Next, these confirmed target gene sequences were used as queries to BLAST against the NCBI GenBank for each target of each species. The first search step obtained target sequences for species from most orders. Then, we selected the obtained target sequences from species with annotated genome as the secondary queries to search against other species within the same order. These two step searches yielded most sequences of four targets in the tested species. For species still without target sequences, we used the target sequences from the closely related species as the tertiary queries to search against the genome of this species.

To collect detoxification gene sequences of different species as comprehensively as possible, genome official gene set (OGS) files for the species were downloaded. Then, we selected all the sequences annotated as “cytochrome P450” or “glutathione S‐transferase” or “carboxyl/cholinesterase”. For some important insect species without published genome OGSs, the detoxification gene sequences were obtained by directly searching against NCBI nucleotide database with terms: (((cytochrome P450) OR glutathione S‐transferase) OR carboxyl/cholinesterase) AND “species name” [Organism].

### 2.3 RNA‐Seq datasets

We searched the NCBI SRA database with the term “insecticide resistance,” yielding a total of 94 RNA‐Seq datasets from 28 populations of 12 insect species. Among these, 51 RNA‐Seq datasets from 15 populations of 6 insect species were submitted with resistance phenotypes. We downloaded the datasets from these populations, including nine RNA‐Seq datasets from three *P. xylostella* populations (CHS, ZZ, and CHR), six RNA‐Seq datasets from two *A. gossypii* populations (NS and KR), 16 RNA‐Seq datasets from four *A. arabiensis* populations (Mozambique, Asendabo, Chewaka, and Tolay), eight RNA‐Seq datasets from three *M. domestica* populations (aabys, KS8S3, and ALHF), six RNA‐Seq datasets from two *L. decemlineata* populations (RS and SS), and six RNA‐Seq datasets from *A. mellifera* for further analysis. CHS was a susceptible population while ZZ and CHR populations were resistant to chlorantraniliprole with a resistance level 42‐fold and 65‐fold (Zhu et al., 2017), respectively. NS was a susceptible population while KR was resistant to neonicotinoids with a resistance level 23.8 to 394‐fold (Hirata et al., 2017). Mozambique (MOZ) was a susceptible population while Asendabo (ASN), Chewaka (CHW), and Tolay (TOL) were resistant to deltamethrin and DDT (Simma et al., 2019). aabys was a susceptible population. KS8S3 was a multi‐resistant population (Reid et al., 2019). And ALHF was resistant to permethrin more than 1,800‐fold compared with aabys population (Li, Reid, et al., 2013). SS was susceptible population while RS was resistant to imidacloprid. Three *A. mellifera* RNA‐Seq datasets were sequenced after selection of imidacloprid, while the other three RNA‐Seq datasets were set as controls.

### 2.4 Preparing FastD inputs

To identify target‐site mutations and detoxification genes associated with resistance phenotypes, clean reads from RNA‐Seq datasets should be mapped to the corresponding target gene sequence and all detoxification gene sequences. For the detection of markers conferring resistance to chlorantraniliprole in *P. xylostella* populations, clean reads were mapped to RyR gene and detoxification gene sequences. For the detection of markers conferring resistance to neonicotinoids in *A. gossypii* populations, clean reads were mapped to nAChR gene and detoxification gene sequences. For the detection of markers conferring resistance to deltamethrin and DDT in *A. arabiensis* populations, clean reads were mapped to VGSC gene and detoxification gene sequences. For the detection of markers conferring resistance to permethrin in *M. domestica* populations, clean reads were mapped to VGSC gene and detoxification gene sequences. For the detection of markers conferring resistance to imidacloprid in *L. decemlineata* populations, clean reads were mapped to nAChR gene and detoxification gene sequences. For the detection of genetic changes after imidacloprid selection in *A. mellifera* populations, clean reads were mapped to nAChR gene and detoxification gene sequences. Generated RNA‐Seq alignments (sam file) from resistant and susceptible populations were utilized as FastD inputs.

### 2.5 Simulation

First, we downloaded the genome annotation of model species, *Drosophila melanogaster* from NCBI. The resistance‐associated genes of four abovementioned targets and three detoxification gene families were selected as reference. To generate artificial target gene reads, we used Flux‐simulator v1.2.1 (Griebel et al., 2012) to simulate the process of RNA sequencing. The simulated reads were then mapped to target gene sequences using bowtie2 (Langdon, 2015) to generate alignment files (sam format). Then, the sam files were transferred into bam format using samtools v1.9 (Li et al., 2009). To simulate variants in alignments, we used BAMsurgeon v1.0 (Ewing et al., 2015) to insert 600 random single‐nucleotide variants with random frequencies into bam files and transferred bam files back to sam format. At last, the sam files were submitted to FastD‐TR, and we called variants and calculated their allele frequencies. We compared the detected variants with the inserted random variants and calculated the sensitivity and specificity of the detected inserted variants. The sensitivity was calculated as the ratio of the number of detected inserted variants to all inserted variants, and the specificity was defined as the ratio of the number of detected inserted variants to the number of all detected variants.

To test the accuracy of differential expression analysis of FastD‐MR, we used polyester v1.24.0 (Frazee et al., 2015) to simulate RNA‐Seq datasets with differential gene expression. We selected 172 detoxification genes in *D. melanogaster* as reference and set the expression fold changes for all genes between two groups each with three replicates. Then, all generated reads from RNA‐Seq datasets were mapped to reference using bowtie2 (Langdon, 2015). The generated alignment files (sam format) were submitted to FastD‐MR to calculate the fold change of each gene between two groups. We compared the detected expression fold changes with the set expression fold changes.

## 3 RESULTS

### 3.1 Resistance‐associated target‐site mutation profiles

**FIGURE 1:**
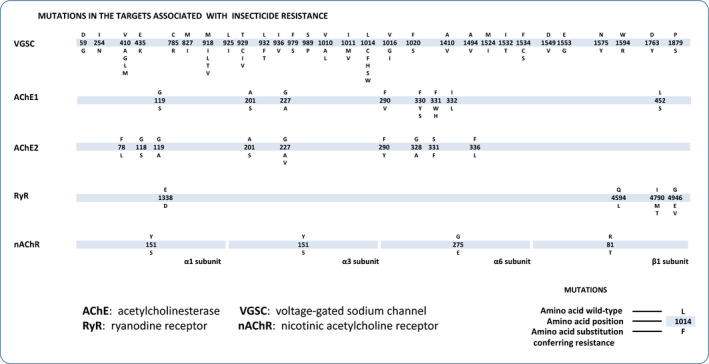
Mutation profiles of four insecticide targets collected by literature mining. The mutation positions in *VGSC*, *AChE* and *RyR* were based on protein sequences of *Musca domestica* (AAB47604.1), *Torpedo Californica* (CAA27169.1) and *P. xylostella* (AET09964.1). In addition, the mutation positions in four subunits of *nAChR*, alpha1, alpha3, alpha6 and beta1, were based on protein sequences of *N. lugens* (AAQ75737.1), *N. lugens* (AAQ75739.1), *Frankliniella occidentalis* (AOT81842.1) and *A. gossypii* (AFH00994.1), respectively

By searching against the NCBI PubMed database, we obtained 440 articles reporting resistance to organophosphates and carbamates associated with target‐site mutations of AChE, 368 articles reporting resistance to pyrethroids associated with target‐site mutations of VGSC, 32 articles reporting resistance to diamides associated with target‐site mutations of RyR, and 81 articles reporting resistance to neonicotinoids associated with target‐site mutations of nAChR. Among these published target‐site mutations, 20 target‐site mutations at 17 sites on AChE were distributed among 36 insect species (Table S1); 46 target‐site mutations at 29 sites on VGSC were distributed among 39 insect species (Table S2); 6 target‐site mutations at 4 sites on RyR were distributed among 4 insect species(Table S3); 4 target‐site mutations at 4 sites on nAChR were distributed among 4 insect species (Table S4). Amino acid positions of all AChE mutations were based on *AChE* of *Torpedo californica* (CAA27169.1); amino acid positions of all VGSC mutations were based on *VGSC* of *Musca domestica* (AAB47604.1); amino acid positions of all *RyR* mutations were based on *RyR* of *P. xylostella* (AET09964.1); amino acid positions of *nAChR* alpha1, alpha3, alpha6, and beta1 subunit mutations were based on *nAChR* alpha1subunit, alpha3 subunit of *Nilaparvata lugens* (AAQ75737.1, AAQ75739.1), alpha6 subunit of *Frankliniella occidentalis* (AOT81842.1), and beta1 subunit of *A. gossypii* (AFH00994.1), respectively (Figure 1).

### 3.2 Resistance‐associated gene sequences

In total, we collected 403 insecticide target gene sequences, including 87 *AChE* sequences (41 ace1 gene sequences and 46 ace2 gene sequences), 71 *VGSC* sequences, 71 *RyR* sequences and 174 *nAChR* sequences (containing 69 alpha1 subunit sequences, 19 alpha3 subunit sequences, 15 alpha6 subunit sequences, and 71 beta1 subunit sequences). All of these gene sequences refer to 82 insect species with *ace* gene sequences belonging to 54 insect species, *VGSC* sequences belonging to 71 insect species, *RyR* sequences belonging to 71 species and *nAChR* sequences belonging to 74 insect species.

Among the 82 insect species, the genome of 71 insects have been published and have annotated OGSs. In total, we extracted 11,356 detoxification gene sequences from the OGS files of 71 insect species. For the remaining 11 species, we obtained 1,309 detoxification gene sequences by searching against NCBI nucleotide database. In total, we obtained 12,665 detoxification gene sequences, including 9,260 P450 gene sequences, 2,188 GST gene sequences and 1,217 CCE gene sequences.

### 3.3 The workflow of FastD program

There are two parts in the FastD program, FastD‐TR (Fast Detection of Target‐site Resistance) to detect target‐site mutations and FastD‐MR (Fast Detection of Metabolic Resistance) to detect overexpressed detoxification genes.

**FIGURE 2:**
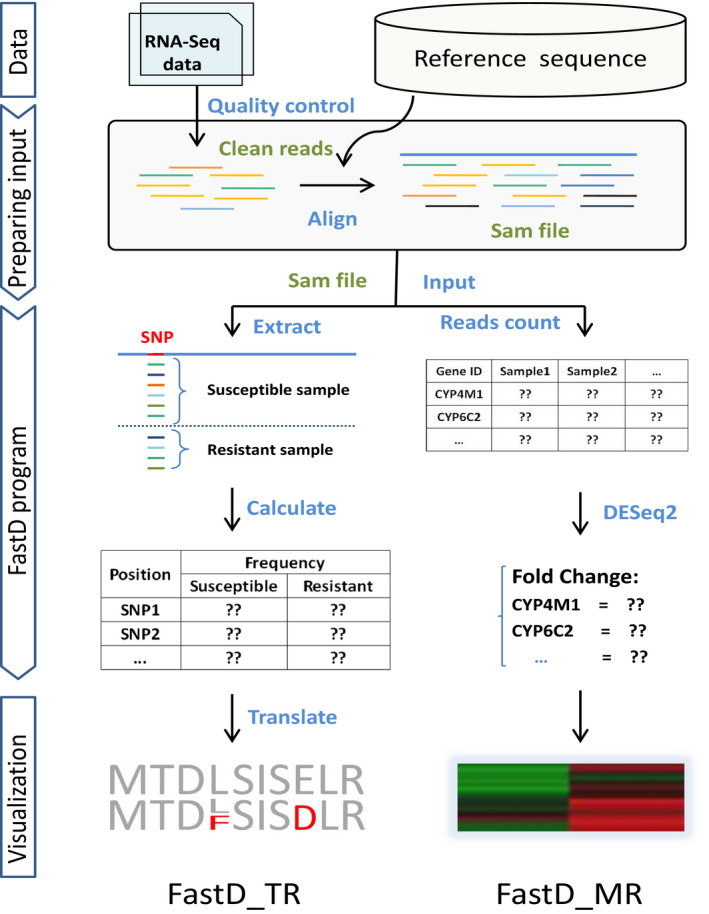
Workflow of the two FastD pipelines, FastD‐TR and FastD‐MR. Fast‐TR was used to detect target‐site mutations and FastD‐MR was used to detect overexpressed detoxification genes. The workflow of FastD‐TR was illustrated in the left part of the figure, and the workflow of FastD‐MR was illustrated in the right part.

The workflow of FastD‐TR consists of six main steps: preprocessing, mapping, SNP calling, differential SNP identification, translation and visualization (Figure 2). To detect previously unknown target‐site mutations associated with insecticide resistance, both the resistant case samples and the susceptible control samples were required for the analysis. Besides, it was optional for the detection of confirmed resistance‐associated target‐site mutations. First, raw reads from RNA‐Seq data of case and control samples should take quality control to filter out adapters and reads with low sequencing quality. The obtained clean reads were then mapped to the target gene sequences using bowtie2 (Langdon, 2015) with additional option, ‐‐no‐unal (filter out unaligned reads), to generate a Sequence Alignment/Map (SAM) file (Li et al., 2009). According to the POS tag of each reads, the nucleotide corresponding to the position on reference gene were extracted for both the case and control samples by a Perl script. For each position including more than one types of corresponding nucleotides and with reads coverage ≥30 was treated as SNP. Next, allele frequency for each SNP was calculated and compared between case and control samples. SNP with allele frequency difference between case and control samples ≥40% in either direction was treated as differential SNP. Then, the codons at differential SNP positions were translated into amino acid residues. Only the nonsynonymous differential SNPs were selected as potential target‐site mutations. An R script named ggseqlogo (Wagih, 2017) was used to visualize the allele distribution in all of the mutation positions.

The workflow of FastD‐MR consists of four main steps: preprocessing, mapping, read count calculation and differential gene expression analysis (Figure 2). The preprocessing step of FastD‐MR was the same as that in FastD‐TR. The obtained clean reads were then mapped to the tested detoxification gene sequences using bowtie2 with additional parameter, ‐‐no‐unal, to generate a SAM file. Read counts of per detoxification gene per sample can be calculated by a Perl Script "rsem‐calculate‐expression" from RSEM software (Li & Dewey, 2011). The reads counts of all genes of all samples were sorted to generated a reads counts matrix. To estimate the expression fold change, read counts matrix was processed by DESeq2 (Love et al., 2014).

### 3.4 Webserver

A webserver (http://www.insect-genome.com/fastd) was constructed to provide online services. The Apache HTTP server (Version 2.4.6) runs on a CentOS Linux 7.4.1708 (core) system. The Web pages were written in HTML and Cascading Style Sheets (CSS). The cDNA sequences of four kinds of insecticide targets and three groups of detoxification genes were stored in a MySQL database (Version 5.7.17). A PHP script was used to call the FastD program when the HTTP server receives the request from a Web client. Both Linux and Windows versions of the FastD standalone software are available for download. The cDNA sequences of the target genes and detoxification genes can also be downloaded from the Webserver.

### 3.5 Evaluation of FastD performance based on simulated data

**FIGURE 3:**
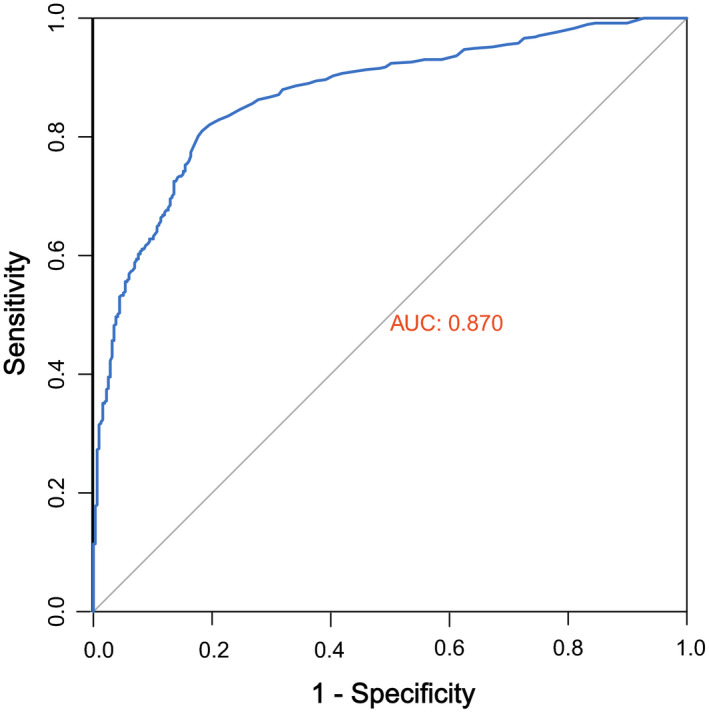
Receiver operating characteristic curve accessing calling performance of FastD‐TR based on the simulated RNA‐Seq data

In the simulated RNA‐Seq reads of target gene, we randomly inserted 600 single‐nucleotide variants into protein coding regions of four target genes and compared the detected variants with the inserted variants. Due to the low coverage of some loci, only 523 variants were inserted successfully. Using FastD‐TR, we detected 469 (89.7%) variants among the inserted variants. We accessed calling performance using AUC (area under curve) in ROC (receiver operating characteristic) curve (Figure 3). ROC with a AUC of 0.870 indicated a reliable calling performance. We compared the detected allele frequencies of detected variants with their set allele frequencies. We found that the allele frequencies calculated by FastD‐TR were highly correlated with their “true” allele frequencies (*R* ^2^ = .834; *p* < 10^–16^). In the RNA‐Seq reads simulation of detoxification gene, we set expression fold changes for 172 detoxification genes between two groups as “true” fold changes. The detected expression fold changes were compared with their true fold changes. We found a high correlation between fold changes calculated by FastD‐MR and fold changes set by polyester (*R* ^2^ = 0.929; *p* < 10^–16^). The simulation results showed the high sensitivity and specificity of FastD.

### 3.6 RyR mutations and overexpressed detoxification genes in diamondback moth

We used FastD‐TR to detect RyR mutations between a resistant population CHR and a susceptible population CHS, and between another resistant population ZZ and CHS. The results showed that there were 12 target‐site mutations detected in CHR versus CHS group, and there were eight target‐site mutations detected in ZZ versus CHS group. Among these target‐site mutations, G4946E, a previously confirmed resistance‐associated target‐site mutation (Troczka et al., 2012), was detected in CHS, CHR and ZZ with frequency of 2.55%, 94.68% and 65.82%, respectively (Table 1). However, FastD‐MR searching showed that no detoxification gene was expressed more than twofold (|log2FoldChange|> 1, *p* value < .01) higher in the CHR compared with CHS. In contrast, six detoxification genes were overexpressed in the ZZ compared with CHS (Table 2). Among these genes, a confirmed resistance‐associated gene *CYP6BG1*, which was reported to be associated with resistance to chlorantraniliprole in *P. xylostella* (Li et al., 2018), had elevated expression levels by 3.3‐fold in ZZ population compared with CHS population.

**TABLE 1:** Target‐site mutations in RyR, with allele frequency and coverage in *Plutella xylostella*‐resistant and susceptible populations

Position	Reference	Variant	CHS		CHR		ZZ		Amino acid substitution
			%	Coverage	%	Coverage	%	Coverage	
1984	A	C	8.41	1,700	82.72	1748	56.68	1796	S662R
2529	C	G	26.79	2016	99.86	2097	92.04	2,311	F843L
5527	C	T	0.33	1533	77.61	1,206			L1843F
6369	T	G	20.01	1,379	95.86	1665	69.94	1,640	F2123L
7131	A	C	0.35	1,443	75.18	1,088			R2377S
9309	A	C	10.12	1562	81.55	1523			L3103F
9642	C	A	12.23	1685	93.38	1,390	72.9	1941	F3214L
10533	G	C	14.6	1,397	87.26	1,484			R3511S
12510	A	C	25.77	947	99.68	949	97.93	964	E4170D
14837	G	A	2.55	1960	94.68	1974	65.82	2,136	G4946E
15492	G	T	10.87	230	56.54	665	56.99	458	E5164D

**TABLE 2:** The overexpressed detoxification genes in ZZ population of *Plutella xylostella* compared with the CHS population

Gene ID	Annotation	FC	Log_2_FC	*SE*	95% CI	*p* Value
XM_011563531.1	*CYP6a2*	10.82	3.44	0.27	2.90–3.97	1.45E−35
XM_011550722.1	*CYP4c21*	4.38	2.13	0.60	0.94–3.32	1.87E−03
XM_011567276.1	*CYP6B6*	3.73	1.90	0.13	1.63–2.16	2.52E−44
XM_011557040.1	*CYP6BG1*	3.27	1.71	0.14	1.43–1.99	1.16E−32
XM_011569337.1	*CYP6k1*	2.35	1.23	0.13	0.97–1.49	3.56E−20
XM_011566337.1	*CYP4C1*	2.08	1.06	0.22	0.61–1.50	1.68E−05

Abbreviations: CI, confidence interval; FC, fold change; *SE*, standard error.

### 3.7 nAChR mutations and overexpressed detoxification genes in cotton aphid

We applied FastD‐TR to detect nAChR mutations between a resistant population KR and a susceptible population NS, showing that only one nAChR mutation was detected on the beta1 of nAChR. A previously confirmed mutation R81T on the beta1 subunit of nAChR was detected with a frequency of 49.85% in KR but not in NS (Table 3). By using FastD‐MR, nine detoxification genes were detected with elevated expression levels more than twofold (|log2FoldChange|> 1, *p* value < .01) in the KR compared with NS (Table 4). Among these genes, *CYP6CY22* and *CYP6CY13*, which were reported to be associated with neonicotinoids resistance (Hirata et al., 2017), had elevated expression levels by 39.61‐ and 22.04‐fold in the resistant KR compared with susceptible NS population, respectively.

**TABLE 3:** The target‐site mutation for R81T on the beta1 subunit of the *nAChR* gene, with allele frequency, the number of variants in reads, and coverage at position “81” in *Aphis gossypii*‐resistant and susceptible populations

Population	Sample	Mutation R81T on beta1 subunit of *nAChR*			
		Variant in reads	Coverage	Frequency (%)	Mean
NS	rep1	0	33	0	0
	rep2	0	77	0	
	rep3	0	45	0	
KR	rep1	24	47	51.06	49.85
	rep2	16	31	51.61	
	rep3	15	32	46.88	

**TABLE 4:** The overexpressed detoxification genes in KR population of *Aphis gossypii* compared with the NS population

Gene ID	Annotation	FC	Log_2_FC	*SE*	95% CI	*p* Value
XM_027986087.1	*CYP6CY22*	39.61	5.31	0.63	4.05–6.57	2.39E−15
XM_027986082.1	*CYP6CY13*	22.04	4.46	0.87	2.73–6.20	2.01E−06
XM_027998534.1	*CYP6a13*	7.00	2.81	0.45	1.91–3.70	1.24E−08
XM_027998535.1	*CYP6a13*	6.61	2.72	0.46	1.80–3.65	5.44E−08
XM_027998540.1	*CYP6a13*	4.85	2.28	0.47	1.34–3.22	8.14E−06
XM_027983025.1	*CYP6a14*	3.92	1.97	0.34	1.30–2.64	5.90E−08
XM_027985966.1	*CYP4C1*	3.21	1.68	0.28	1.12–2.25	4.48E−08
XM_027993601.1	*CYP6a13*	2.88	1.53	0.29	0.95–2.11	1.25E−06
XM_027987563.1	*CYP6a13*	2.77	1.47	0.26	0.95–1.99	1.47E−07

Abbreviations: CI, confidence interval; FC, fold change; *SE*, standard error.

### 3.8 VGSC mutations and overexpressed detoxification genes in *A. arabiensis*

We applied FastD‐TR to detect VGSC mutations in three comparisons, resistant population ASN versus susceptible population MOZ, resistant population CHW versus susceptible population MOZ and resistant population TOL versus susceptible population MOZ groups. Only one previously confirmed VGSC mutation, L1014F, was detected in TOL versus MOZ group. Next, we used FastD‐TR to detect the previously confirmed mutation for all four populations. The result showed that VGSC mutation L1014F were detected in ASN, CHW and TOL populations with 0.25, 0.21 and 0.53 frequencies, respectively, while not detected in susceptible population MOZ (Table 5), by using FastD‐MR to detect overexpressed more than twofold (|log2FoldChange|> 1, *p* value < .01) detoxification genes in the abovementioned three comparisons. The results showed that 23 overexpressed genes were detected in ASN population compared with MOZ population, 25 overexpressed genes were detected in CHW population compared with MOZ population and 26 overexpressed genes were detected in TOL compared with MOZ population. Among these overexpressed genes, 13 genes were overexpressed commonly in three resistant populations compared with susceptible population (Table 6). Among these genes, *CYP6P3* and *CYP6M2* were reported to be associated with pyrethroids resistance in *A. gambiae* (Müller et al., 2008; Stevenson et al., 2011); *CYP6P4* and *CYP4G16* were related to pyrethroids resistance in *A. arabiensis* (Ibrahim et al., 2016; Jones et al., 2013). Comparing resistant ASN, CHW and TOL populations with susceptible population MOZ, *CYP6P3* had elevated expression levels by 4.38‐, 21.95‐ and 6.03‐fold, respectively; *CYP6M2* had elevated expression levels by 6.5‐, 14.13‐ and 6.06‐fold, respectively; *CYP6P4* had elevated expression levels by 4.12‐, 6.45‐ and 6.27‐fold, respectively; *CYP4G16* had elevated expression levels by 2.38‐, 2.35‐ and 2.83‐fold, respectively.

**TABLE 5:** The target‐site mutation for L1014F on VGSC, with allele frequency, the number of variants in reads and coverage at position “1014” in *Anopheles arabiensis* resistant and susceptible populations

Population	Sample	Mutation L1014F in *VGSC*		
		Variants in reads	Coverage	Frequency (%)
MOZ	Pool	0	62	0
ASN	Pool	8	32	25
CHW	Pool	8	38	21.1
TOL	Pool	20	38	52.6

**TABLE 6:** The comparisons of overexpressed detoxification genes in the ASN versus MOZ, CHW versus MOZ and TOL versus MOZ groups, with fold change >2 and *p* value <.01

Gene ID	Annotation	ASN vs. MOZ		CHW vs. MOZ		TOL vs. MOZ	
		FC	*p* Value	FC	*p* Value	FC	*p* Value
AARA015787‐RA	*CYP6P3*	4.38	1.46E−10	21.95	1.96E−11	6.03	2.99E−12
AARA015765‐RA	*GSTD10*	15.45	8.77E−07	7.28	6.77E−03	11.13	3.40E−09
AARA015644‐RA	*CYP6M2*	6.50	1.26E−16	14.13	6.10E−24	6.06	3.91E−25
AARA015719‐RA	*GSTU2*	7.56	1.53E−06	3.61	7.27E−03	4.25	1.67E−03
AARA002507‐RA	*CYP9K1*	4.08	6.59E−09	6.50	6.72E−13	4.04	1.87E−08
AARA015789‐RA	*CYP6P4*	4.12	5.67E−11	6.45	4.90E−15	6.27	3.69E−22
AARA011200‐RA	*CYP4H17*	3.34	8.62E−09	5.80	2.06E−21	4.47	7.36E−16
AARA011201‐RA	*CYP4H18*	5.03	5.36E−12	3.03	3.50E−05	5.59	5.89E−14
AARA015729‐RA	*GSTE1*	5.35	9.13E−16	3.13	8.24E−09	4.22	1.88E−24
AARA015862‐RA	*CYP6AG2*	3.78	2.16E−19	4.39	2.18E−30	4.17	2.13E−31
AARA001582‐RA	*CCE*	2.26	1.80E−06	2.43	1.36E−05	3.34	2.78E−19
AARA011787‐RA	*CYP4G16*	2.38	6.94E−05	2.35	1.74e−04	2.83	1.56E−06
AARA015764‐RA	*GSTD3*	2.78	7.71E−09	2.24	1.55E−07	2.76	2.25E−18

Abbreviation: FC, fold change.

### 3.9 VGSC mutations and overexpressed detoxification genes in house fly

We applied FastD‐TR to detect potential VGSC mutations in two comparisons, resistant population KS8S3 versus susceptible population aabys, resistant population ALHF versus susceptible population aabys. The results showed that five VGSC mutations including a previously known mutation L1014F were detected between ALHF and aabys populations while only one appeared in KS8S3 and aabys populations (Table 7). Next, we used FastD‐TR to detect the previously confirmed mutation for all three populations. The result showed that VGSC mutation L1014F were detected in KS8S3 and ALHF populations with frequency of 15.09% and 99.32%, respectively, while not detected in susceptible population aabys, by using FastD‐MR to detect overexpressed more than twofold (|log2FoldChange|> 1, *p* value < .01) detoxification genes in the abovementioned two comparisons. The results showed that 48 overexpressed genes were detected in KS8S3 population compared with aabys population and 55 overexpressed genes were detected in ALHF population compared with aabys population. Among these overexpressed genes, 16 genes were overexpressed commonly in two resistant populations compared with susceptible population (Table 8). The pyrethroids resistance marker gene *CYP6D1* was not among the overexpressed genes.

**TABLE 7:** Target‐site mutations on VGSC, with allele frequency and coverage in *Musca domestica*‐resistant and susceptible populations

Position	Reference	Variant	aabys		KS8S3		ALHF		Amino acid substitution
			%	Coverage	%	Coverage	%	Coverage	
3040	C	T	0	603	15.09	106	99.32	147	L1014F
5656	C	A	32.14	1,565			100.00	178	R1886S
5818	C	G	0.12	1,731			99.24	263	R1940G
6088	A	G	3.86	1,243			97.47	79	T2030A
6155	G	C	35.36	1,281	98.58	211	100.00	133	A2052G

**TABLE 8:** The comparisons of overexpressed detoxification genes in the KS8S3 versus aabys and ALHF versus aabys groups, with fold change >2 and *p* value <.01

Gene ID	Annotation	KS8S3 vs. aabys		ALHF vs. aabys	
		FC	*p* Value	FC	*p* Value
XM_011296811.2	*CYP313a4*	166.55	1.09E−76	295.47	8.83E−83
XM_020037027.1	*CYP6a14*	268.52	1.69E−99	4.3	2.13E−03
XM_005180691.3	*CCE−6*	258.78	9.55E−08	42.66	1.62E−19
XM_005186599.3	*GST1*	61.28	5.04E−05	92.33	4.31E−05
NM_001309038.1	*CYP4d14*	6.31	8.87E−08	70.06	8.01E−27
XM_005175041.2	*CYP313a4*	53.61	2.11E−35	69.66	9.17E−31
XM_005186271.3	*CYP4e2*	40.65	2.47E−22	42.87	1.18E−18
XM_011295499.2	*GST1*	16.2	2.70E−03	24.91	3.22E−04
XM_005175565.3	*CYP6a2*	8.86	1.17E−08	19.8	1.59E−13
XM_020039254.1	*CYP6a21*	2.9	8.81E−06	8.86	3.87E−21
XM_005180042.3	*GST4*	4.6	1.41E−06	8.61	2.38E−10
XM_005190450.3	*CYP6g1*	4.72	1.56E−03	5.8	6.32E−04
XM_011293819.2	*CYP313b1*	3.25	2.07E−08	5.78	6.34E−11
XM_011294527.2	*CYP28d1*	3.14	4.14E−03	5.17	1.62E−05
XM_011293587.2	*CCE‐6*	2.33	7.19E−04	3.94	7.01E−10
XM_020039255.1	*CYP6a22*	2.74	3.52E−03	3.56	8.02E−05

Abbreviation: FC, fold change.

### 3.10 Overexpressed detoxification genes in Colorado potato beetle

We detected nAChR mutations between a resistant population and a susceptible population in Colorado potato beetle. There was no nAChR mutation found both in the resistant and susceptible populations. By using FastD‐MR, 17 detoxification genes, including 13 P450s and 4 CCEs, were detected with elevated expression levels more than twofold (|log2FoldChange|> 1, *p* value < .01) in the resistant compared with susceptible population with fold changes ranging from 20.07 to 2.17 (Table 9).

**TABLE 9:** The overexpressed detoxification genes in resistant population of *Leptinotarsa decemlineata* compared with the susceptible population

Gene ID	Annotation	FC	Log_2_FC	*SE*	95% CI	*p* Value
XM_023162345.1	*CYP9e2*	21.07	4.40	0.60	3.19–5.61	3.38E−13
XM_023162346.1	*CYP9e2*	16.41	4.04	0.60	2.83–5.25	2.50E−11
XM_023165848.1	*CYP4C1*	12.68	3.66	0.20	3.26–4.07	1.64E−72
XM_023169053.1	*CYP6a13*	11.40	3.51	1.16	1.18–5.84	2.56E−03
XM_023166585.1	*CYP6k1*	7.51	2.91	0.72	1.47–4.34	4.97E−05
XM_023165075.1	*CYP6a14*	5.74	2.52	0.80	0.93–4.11	1.58E−03
XM_023172322.1	*CCE6*	4.59	2.20	0.34	1.51–2.89	1.61E−10
XM_023166383.1	*CYP12a5*	3.84	1.94	0.19	1.56–2.33	4.69E−24
XM_023160597.1	*CCE6*	3.44	1.78	0.25	1.28–2.29	1.60E−12
XM_023168455.1	*CYP4C1*	3.34	1.74	0.50	0.74–2.74	5.16E−04
XM_023168661.1	*CCE5A*	3.30	1.72	0.29	1.14–2.31	4.07E−09
XM_023172620.1	*CYP301a1*	2.98	1.58	0.29	1.00–2.16	5.47E−08
XM_023158724.1	*CYP4c3*	2.51	1.33	0.35	0.62–2.03	1.70E−04
XR_002722815.1	*CYP6a13*	2.49	1.32	0.22	0.88–1.75	1.61E−09
XM_023168255.1	*CYP6a20*	2.49	1.31	0.26	0.79–1.83	4.08E−07
XM_023166623.1	*CYP6a23*	2.42	1.28	0.24	0.80–1.75	8.89E−08
XM_023172365.1	*CCE6*	2.17	1.12	0.18	0.76–1.47	3.66E−10

Abbreviations: CI, confidence interval; FC, fold change; *SE*, standard error.

### 3.11 Overexpressed detoxification genes in honey bee

For honey bee, there were no corresponding RNA‐Seq datasets from resistant populations available. We applied FastD to detect the genetic changes of honey bee after selection of imidacloprid. Using FastD‐TR, there was no target‐site mutation detected both in selected samples or control samples. While four detoxification genes were detected with overexpression in selected sample compared with control sample using FastD‐MR (Table 10).

**TABLE 10:** The overexpressed detoxification genes in imidacloprid selected samples of *Apis mellifera* compared with the control samples

Gene ID	Annotation	FC	Log_2_FC	*SE*	95% CI	*p* Value
XM_006562301.3	*CYP9e2*	3.08	1.62	0.47	0.68–2.56	5.7E−04
XM_006562300.3	*CYP9e2*	2.88	1.52	0.39	0.75–2.30	7.65E−05
XM_006557827.3	*CYP306a1*	2.37	1.24	0.46	0.32–2.16	6.95E−03
XM_016915819.2	*CYP306a1*	2.28	1.19	0.39	0.4–1.98	2.59E−03

Abbreviations: CI, confidence interval; FC, fold change; *SE*, standard error.

## 4 DISCUSSION

Insecticide resistance monitoring is the key to sustain insecticide‐mediated control efficiency. Molecular detecting assays can be used to detect resistant markers accurately at early stages to avoid resistance evolution (Network, 2016). Target‐site resistance, which is mainly caused by target‐site mutations, and metabolic resistance, which is mainly caused by overexpressed detoxification genes, are the two main mechanisms of insecticide resistance (Ffrench‐Constant, 2013). The detection of these two kinds of resistance can well reveal the mechanism of resistance of insect pest populations. PCR‐based target‐site mutation detection assays rely on genotyping individuals one by one within an insect pest population and are not only time‐consuming, but also result in a high false‐positive rate (Blais et al., 2015; Hirayama et al., 2010). The DNA microarray which used to detect differentially expressed detoxification genes are inefficient and complex, because of the demand for prerequisite knowledge of the reference sequences, low resolution of expression level, and background signals (Kogenaru et al., 2012; Mantione et al., 2014). RNA‐Seq sequences the transcription products of pooled samples of insect pest populations and can obtain the SNP information in gene expressed regions as well as provide gene expression level comparison (De Wit et al., 2015). More and more researchers have adopted RNA‐Seq as a method to study resistance mechanisms and detect resistant markers (David et al., 2014; Faucon et al., 2017; Mamidala et al., 2012).

ACE was a tool developed to detect previously known target‐site mutations in organophosphates and carbamates target AChE from RNA‐Seq data (Guo et al., 2017). Compared with ACE, FastD was developed to detect the target‐site mutations on four targets and overexpressed detoxification genes. The resistance estimation was more comprehensive. Besides, by comparing RNA‐Seq data from resistant and susceptible populations, the FastD program can also identify the new target‐site mutations. The webserver of the FastD program using SAM files as input which can analyze the samples more quickly than ACE using fastq files as input. With these characteristics, FastD program offers a wider range of applications and greater value.

As a proof of concept, FastD program was used to detect the resistance markers and identify candidate markers of six insects, *P. xylostella*, *A. gossypii*, *A. arabiensis*, *M. domestica*, *L. decemlineata* and *A. mellifera*. The resistance of insect populations can be well estimated by these resistant markers via FastD program. The resistance‐associated target‐site mutations, RyR mutation G4946E, nAChR mutation R81T, and VGSC mutation L1014F all exhibited higher frequency in resistant populations compared with susceptible populations. The expression of resistance‐associated detoxification genes, *CYP6BG1*, *CYP6CY22*, *CYP6CY13*, *CYP6P3*, *CYP6M2*, *CYP6P4* and *CYP4G16* also exhibited more than two fold higher in resistant populations compared with susceptible populations. In addition, other 10 RyR mutations in *P. xylostella* and four VGSC mutations in *M. domestica* with frequency difference >40% between resistant and susceptible populations and other five detoxification genes in *P. xylostella*, seven detoxification genes in *A. gossypii*, 13 detoxification genes in *A. arabiensis*, and 16 detoxification genes in *M. domestica* overexpressed in resistant populations compared with susceptible populations were worthy further validation to serve as novel resistance markers.

As a tool to detect resistant markers to monitor the emergence and development of insecticide resistance and to identify candidate mutations and genes as novel markers from RNA‐Seq data, there are still some limitations. We plan to improve the following areas in the future. First, insecticide resistance with the polygene inheritance model is also associated with other important mechanisms, especially the detoxification gene amplification. Due to the limitation of RNA‐Seq technique, gene amplification cannot be identified by FastD‐MR. We plan to add new function to identify gene amplification based on genome resequencing data. Second, the accuracy of mutation frequency calculated by FastD‐TR is limited by the fact that RNA‐Seq reads from pooled sample have potentially different levels of contribution from each insect sample and allele. Therefore, we recommend users to use larger number of individuals sampled in pool to get more accurate result. Third, the resistance level is determined empirically based on detected resistant markers by the FastD program. More quantitative relationships between the resistant markers and resistance are critical and could be established with machine learning methods. Fourth, aside from insecticide resistance, resistance in other pests (herbicide resistance and fungicide resistance) is also associated with target‐site mutations and overexpressed detoxification genes (Bohnert et al., 2019; Li, Fang, et al., 2013). Estimating the resistance to herbicide and fungicide will be added in the next version of FastD program.

## CONFLICT OF INTEREST

The authors declare that they have no competing interests.

## AUTHOR CONTRIBUTION

**Longfei Chen:** Data curation (lead); Investigation (lead); Methodology (lead); Resources (equal); Software (equal); Writing‐original draft (lead). **Kun Lang:** Data curation (supporting); Formal analysis (lead); Investigation (equal); Resources (lead); Software (supporting). **Yang Mei:** Data curation (supporting); Software (supporting); Validation (supporting); Visualization (supporting). **Zhenmin Shi:** Data curation (supporting); Formal analysis (supporting); Software (supporting). **Kang He:** Investigation (supporting); Resources (supporting); Writing‐review & editing (supporting). **Fei Li:** Conceptualization (lead); Funding acquisition (lead); Project administration (lead); Supervision (lead); Writing‐review & editing (lead). **Huamei Xiao:** Data curation (equal); Methodology (lead); Project administration (lead); Software (equal); Supervision (equal); Visualization (equal); Writing‐review & editing (supporting). **Gongyin Ye:** Conceptualization (supporting); Data curation (supporting); Project administration (supporting); Supervision (supporting); Writing‐review & editing (supporting). **Zhaojun Han:** Conceptualization (supporting); Project administration (supporting); Supervision (supporting); Validation (supporting); Writing‐review & editing (supporting).

## Supporting information

Table S1

Table S2

Table S3

Table S4

## Data Availability

The FastD program, the online version and standalone version, installation instructions, resistance‐related gene sequences, and a step‐by‐step example on how to use were freely available at the web server: http://www.insect-genome.com/fastd. The RNA‐Seq datasets used in this study were publicly available (NCBI SRA database): under the accession of SRP095967, DRP003736, SRP164719, SRP014193, SRP080926, and SRP140405.
