# Supplementary material for: FastD: Fast detection of insecticide target‐site mutations and overexpressed detoxification genes in insect populations from RNA‐Seq data
Source: Ecol Evol. 2020 Nov 21;10(24):14346–58. doi: 10.1002/ece3.7037 (PMC7771117; doi:10.1002/ece3.7037)
Supplement: Supplementary file 1 — Table S1 [file ECE3-10-14346-s001.docx]

Table S1 Resistance-associated mutations in *AChE1* and *AChE2* of insects

| **Gene** | **Position^a^** | **Insect species** | **Mutation** | **References** |
| --- | --- | --- | --- | --- |
| ***AChE1*** | 85 | *Aphis gossypii* | S146N | Shang Q. et al., Environ Toxicol (2014) |
|  | 119 | *Aedes aegypti* | G119S | Muthusamy R, Shivakumar MS. J Vector Borne Dis (2015) |
|  |  | *Anophele albimanus* | G119S | Weill et al., Nature (2003) |
|  |  | *Anopheles coluzzii* | G119S | Essandoh J. et al., Malar J (2013) |
|  |  | *Anopheles funestus* | G119S | Djouaka R. et al., PLoS One (2011) |
|  |  | *Anopheles gambiae* | G119S | Weill et al., Nature (2003) |
|  |  | *Anopheles stephensi* | G119S | Soltani A. et al., J Arthropod Borne Dis (2014) |
|  |  | *Culex pipiens* | G119S | Alout H. et al., Insect Biochem Mol Biol (2009) |
|  |  | *Culex pipiens quinquefasciatus* | G119S | Misra BR, Gore M. J Med Entomol (2015) |
|  |  | *Culex vishnui* | G119S | Alout H. et al., J Med Entomol (2007) |
|  |  | *Lutzomyia longipalpis* | G119S | Coutinho-Abreu IV. et al., J Med Entomol (2007) |
|  |  | *Phlebotomus papatasi* | G119S | Temeyer KB. et al., Parasit Vectors (2014) |
|  |  | *Nilaparvata lugens* | G119A | Deok Ho Kwon.et al., Pesticide Biochemistry and Physiology (2012) |
|  | 201 | *Culex pipiens quinquefasciatus* | A328S | Zhao M.et al., PLoS One (2014) |
|  |  | *Apolygus lucorum* | A216S | Shuwen Wu. et al., Insect Biochem Mol Biol (2015) |
|  |  | *Therioaphis trifolii* | A302S | AlSuhaibani E. et al., Bull Entomol Res (2015) |
|  |  | *Bombyx mori* | A303S | Wang JM. et al., Mol Biol Rep (2014 |
|  |  | *Chilo auricilius* | A314S | Luo GH. et al., J Econ Entomol (2015) |
|  |  | *Chilo suppressalis* | A314S | Jiang X. et al., Biochem Biophys Res Commun (2009) |
|  |  | *Plutella xylostella* | A298S | Yeh SC. et al., Pestic Biochem Physiol (2014) |
|  |  | *Spodoptera frugiperda* | A201S | Carvalho RA. et al., PLoS One (2013) |
|  | 216 | *Aphis gossypii* | G292R | Shang Q. et al., Environ Toxicol (2014) |

**Table S1.** Continued

| **Gene** | **Position^a^** | **Insect species** | **Mutation** | **References** |
| --- | --- | --- | --- | --- |
| ***AChE1*** | 227 | *Bombyx mori* | G329A | Wang JM. et al., Mol Biol Rep (2014) |
|  |  | *Plutella xylostella* | G324A | Yeh SC.et al., Pestic Biochem Physiol (2014) |
|  |  | *Spodoptera frugiperda* | G227A | Carvalho RA.et al., PLoS One (2013) |
|  | 228 | *Rhopalosiphum padi* | S329P | Chen MH. et al., Genome (2007) |
|  | 268 | *Aphis gossypii* | P348S | Shang Q. et al., Environ Toxicol (2014) |
|  | 290 | *Culex pipiens* | F290V | Alout H. et al., Insect Biochem Mol Biol (2009) |
|  |  | *Chilo suppressalis* | F402V | Chang C. et al., Pest Manag Sci (2014) |
|  |  | *Cydia pomonella* | F388V | Reyes M. et al., Bull Entomol Res (2009) |
|  |  | *Plutella xylostella* | F386F/V | Yeh SC. et al., Pestic Biochem Physiol (2014) |
|  |  | *Spodoptera frugiperda* | F290V | Carvalho RA. et al., PLoS One (2013) |
|  | 330 | *Nilaparvata lugens* | F/Y330S | Deok Ho Kwon. et al., Pesticide Biochemistry and Physiology (2012) |
|  | 331 | *Culex Tritaeniorhynchus* | F455W | Misra BR, Gore M. J Med Entomol (2015) |
|  |  | *Aphis gossypii* | S409 F | Cuamba N. et al., PLoS One (2010) |
|  |  | *Bemisia tabaci* | F392W | Zhang NN. et al., J Insect Sci (2012) |
|  |  | *Nilaparvata lugens* | F331H | Deok Ho Kwon. et al., Pesticide Biochemistry and Physiology (2012) |
|  |  | *Therioaphis trifolii* | S431F | AlSuhaibani E. et al., Bull Entomol Res (2015) |
|  | 332 | *Nilaparvata lugens* | I332L | Deok Ho Kwon. et al., Pesticide Biochemistry and Physiology (2012) |
|  | 441 | *Plutella xylostella* | A441G | Ji Hyung Baek. et al., Pesticide Biochemistry and Physiology (2005) |
|  | 451 | *Aphis gossypii* | L532P | Shang Q. et al., Environ Toxicol (2014) |
|  | 452 | *Bombyx mori* | L554S | Wang JM. et al., Mol Biol Rep (2014) |
|  | —— | *Chilo suppressalis* | H668P | Chang C. et al., Pest Manag Sci (2014) |
|  | —— | *Chilo suppressalis* | R667Q | Dai SM. et al., Pest Manag Sci (2015) |

**Table S1.** Continued

| **Gene** | **Position^a^** | **Insect species** | **Mutation** | **References** |
| --- | --- | --- | --- | --- |
| ***AChE2*** | 78 | *Drosophila melanogaster* | F115(78)S | Mutéro et al., Proc. Natl. Acad. Sci. USA (1994) |
|  | 78 | *Aphis gossypii* | F139L | Fei Li, Zhaojun Han. Insect Biochem Mol Biol (2004) |
|  | 79 | *Oomyzus sokolowskii* | E115 L | Zhuang HM. et al., Mol Biol Rep (2014) |
|  | 118 | *Nilaparvata lugens* | Gly185Ser | Yang Z. et al., J Insect Sci (2010) |
|  | 119 | *Aphis gossypii* | G221A | Lokeshwari D. et al., J Econ Entomol (2016) |
|  | 129 | *Bactrocera dorsalis* | I214V | Hsu JC. et al., Insect Biochem Mol Biol (2006) |
|  |  | *Bactrocera oleae* | I214V | Başkurt SI. et al., Acta Biol Hung (2011) |
|  |  | *Cochliomyia hominivorax* | I298V | da Silva NM. et al., Vet Parasitol (2011) |
|  |  | *Drosophila melanogaster* | I119(129)T | Mutéro et al., Proc. Natl. Acad. Sci. USA (1994) |
|  |  | *Drosophila melanogaster* | I119(129)V | Mutéro et al., Proc. Natl. Acad. Sci. USA (1994) |
|  | 150 | *Musca domestica* | V180(150)L | Walsh et al., Biochem. J. (2001) |
|  |  | *Musca domestica* | V260L | Başkurt S. et al., J Vector Ecol（2011） |
|  | 201 | *Aphis gossypii* | A302S | Lokeshwari D. et al., J Econ Entomol (2016) |
|  | 227 | *Cochliomyia hominivorax* | G401A | da Silva NM. et al., Vet Parasitol (2011) |
|  |  | *Drosophila melanogaster* | G303A | Mutéro et al., Proc. Natl. Acad. Sci. USA (1994) |
|  |  | *Musca domestica* | G342A/V | Başkurt S. et al., J Vector Ecol (2011) |
|  |  | *Haematobia irritans* | G262A | Temeyer KB. et al., J Med Entomol (2012) |
|  |  | *Drosophila melanogaster* | G265A | Menozzi P. et al., BMC Evol Biol (2004) |
|  | 238 | *Leptinotarsa decemlineata* | S291G | Clark JM. et al., Pest Manag Sci (2001) |
|  | 271 | *Alphitobius diaperinus* | A271S | Kozaki T. et al., Arch Insect Biochem Physiol (2008) |
|  | 290 | *Drosophila melanogaster* | F368(288)Y | Mutéro et al., Proc. Natl. Acad. Sci. USA (1994) |

**Table S1.** Continued

| **Gene** | **Position^a^** | **Insect species** | **Mutation** | **References** |
| --- | --- | --- | --- | --- |
| ***AChE2*** | 290 | *Cochliomyia hominivorax* | F466Y | da Silva NM. et al., Vet Parasitol (2011) |
|  |  | *Drosophila melanogaster* | F330Y | Menozzi P. et al., BMC Evol Biol (2004) |
|  |  | *Musca domestica* | F327(288)Y | Walsh et al., Biochem. J. (2001) |
|  |  | *Musca domestica* | Y407F | Başkurt S. et al., J Vector Ecol(2011) |
|  |  | *Rhopalosiphum padi* | F368(290)L | Chen MH. et al., Genome (2007) |
|  | 328 | *Ceratitis capitata* | G328A | Elfekih S. et al., J Econ Entomol (2014) |
|  |  | *Drosophila melanogaster* | G368A | Menozzi P. et al., BMC Evol Biol (2004) |
|  |  | *Musca domestica* | G365(328)A | Walsh et al., Biochem. J. (2001) |
|  | 331 | *Aphis gossypii* | S431F | Lokeshwari D. et al., J Econ Entomol (2016) |
|  |  | *Myzus persicae* | S431F | Cassanelli S. et la., Pest Manag Sci (2005) |
|  | 336 | *Oomyzus sokolowskii* | F394 L | Zhuang HM. et al., Mol Biol Rep (2014) |
|  | 356 | *Rhopalosiphum padi* | V435(356)A | Chen MH. et al., Genome (2007) |
|  | 365 | *Oomyzus sokolowskii* | K424R | Zhuang HM. et al., Mol Biol Rep (2014) |
|  | 396 | *Bactrocera dorsalis* | G488S | Hsu JC. et al., Insect Biochem Mol Biol (2006) |
|  |  | *Bactrocera oleae* | G488S | J. G. Vontas. et al., Insect Mol. Biol (2002) |
|  | 484 | *Aphis gossypii* | K585R | Shang Q. et al., Environ Toxicol (2014) |
|  | 510 | *Helicoverpa armigera* | A585T | Ren X. et al., Arch. Insect Biochem. Physiol. (2002) |
|  | —— | *Bactrocera dorsalis* | Q643R | Hsu JC. et al., Insect Biochem Mol Biol (2006) |

Position^a^: numbering according to *ACHE* of *Torpedo californica*.
