# Supplementary material for: FastD: Fast detection of insecticide target‐site mutations and overexpressed detoxification genes in insect populations from RNA‐Seq data
Source: Ecol Evol. 2020 Nov 21;10(24):14346–58. doi: 10.1002/ece3.7037 (PMC7771117; doi:10.1002/ece3.7037)
Supplement: Supplementary file 2 — Table S2 [file ECE3-10-14346-s002.docx]

Table S2 Resistance-associated mutations in *VGSC* of insects

| **Gene** | **Position^a^** | **Insect species** | **Mutation** | **References** |
| --- | --- | --- | --- | --- |
| ***VGSC*** | 59 | *Blattella germanica* | D59G | Liu Z. et al., Insect Biochem Mol Biol (2002) |
|  | 99 | *Culex quinquefasciatus* | A99S | Li T. et al., Sci Rep (2012) |
|  | 254 | *Drosophila melanogaster* | I254N | Pittendrigh B. et al., Mol Gen Genet (1997) |
|  | 410 | *Cimex lectularius* | V410L | Yoon KS. et al., J Med Entomol (2008) |
|  |  | *Helicoverpa zea* | V421A/M/G | Hopkins BW. et al., Insect Biochem Mol Biol (2010) |
|  |  | *Heliothis virescens* | V410M | Park Y. et al., Biochem Biophys Res Commun (1997) |
|  | 435 | *Blattella germanica* | E435K | Liu Z. et al., Insect Biochem Mol Biol (2002) |
|  | 785 | *Blattella germanica* | C785R | Liu Z. et al., Insect Biochem Mol Biol (2002) |
|  | 827 | *Pediculus capitis* | M827I | SupYoon K. et al., Insect Biochem Mol Biol (2008) |
|  |  | *Pediculus corporis* | M827I | Drali R. et al., J Clin Microbiol (2012) |
|  | 918 | *Haematobia irritans* | M918T | Guerrero FD. et al., Insect Biochem Mol Biol (1997) |
|  |  | *Musca domestica* | M918T | Williamson MS. et al., Mol Gen Genet (1996) |
|  |  | *Tuta absoluta* | M918T | Haddi K. et al., Insect Biochem Mol Biol (2012) |
|  |  | *Bemisia tabaci* | M918V | Morin S. et al., Insect Biochem Mol Biol (2002) |
|  |  | *Cimex hemipterus* | M918I | Dang K. et al., Pest Manag Sci (2015) |
|  |  | *Myzus persicae* | M918T/L | Panini M. et al., Pest Manag Sci (2015) |
|  |  | *Plutella xylostella* | M918I | Sonoda S. et al., Insect Biochem Mol Biol (2008) |
|  |  | *Thrips tabaci* | M918T/L | Toda S. et al., J Econ Entomol (2009) |
|  | 925 | *Bemisia tabaci* | L925I | Morin S. et al., Insect Biochem Mol Biol (2002) |
|  |  | *Cimex lectularius* | L925I | Yoon KS. et al., J Med Entomol (2008) |
|  |  | *Triatoma infestans* | L925I | Capriotti N. et al., PLoS Negl Trop Dis (2014) |

**Table S2.** Continued.

| **Gene** | **Position^a^** | **Insect species** | **Mutation** | **References** |
| --- | --- | --- | --- | --- |
| ***VGSC*** | 929 | *Sitophilus zeamais* | T929I | Araújo RA. et al., Insect Mol Biol (2011) |
|  |  | *Tuta absoluta* | T929I | Haddi K. et al., Insect Biochem Mol Biol (2012) |
|  |  | *Bemisia tabaci* | T929V | Alon M. et al., Insect Biochem Mol Biol (2006) |
|  |  | *Plutella xylostella* | T929I | Schuler. et al., (1998) |
|  |  | *Spodoptera frugiperda* | T929I | Carvalho RA. et al., PLoS One (2013) |
|  |  | *Pediculus capitis* | T929I | SupYoon K. et al., Insect Biochem Mol Biol (2008) |
|  |  | *Pediculus corporis* | T929I | Drali R. et al., J Clin Microbiol (2012) |
|  |  | *Frankliniella occidentalis* | T929C | Forcioli. et al., (2002) |
|  |  | *Thrips tabaci* | T929I | Toda S. et al., J Econ Entomol (2009) |
|  | 932 | *Aedes aegypti* | L932T | Brengues C. et al., Med Vet Entomol (2003) |
|  |  | *Spodoptera frugiperda* | L932F | Carvalho RA. et al., PLoS One (2013) |
|  |  | *Pediculus capitis* | L932F | SupYoon K. et al., Insect Biochem Mol Biol (2008) |
|  |  | *Pediculus corporis* | L932F | Drali R. et al., J Clin Microbiol (2012) |
|  | 936 | *Helicoverpa zea* | I951V | Hopkins BW. et al., Insect Biochem Mol Biol (2010) |
|  | 989 | *Aedes aegypti* | S989P | Srisawat R. et al., Southeast Asian J Trop Med Public Health (2012) |
|  | 1010 | *Anopheles culicifacies* | V1010L | Singh OP. et al., Malar J (2010) |
|  |  | *Thrips tabaci* | V1010A | Wu M. et al., Pest Manag Sci (2014) |
|  | 1011 | *Aedes aegypti* | I1011M/V | Brengues C. et al., Med Vet Entomol (2003) |
|  | 1014 | *Blattella germanica* | L1014F | Liu Z. et al., Insect Biochem Mol Biol (2002) |
|  |  | *Leptinotarsa decemlineata* | L1014F | Lee. et al., (1999) |

**Table S2.** Continued.

| **Gene** | **Position^a^** | **Insect species** | **Mutation** | **References** |
| --- | --- | --- | --- | --- |
| ***VGSC*** | 1014 | *Meligethes aeneus* | L1014F | Zimmer CT. et al., Insect Mol Biol (2014) |
|  |  | *Psylliodes chrysocephala* | L1014F | Zimmer CT. et al., Pestic Biochem Physiol (2014) |
|  |  | *Anopheles albimanus* | L1014F/C | Lol JC. et al., Parasit Vectors (2013) |
|  |  | *Anopheles arabiensis* | L1014F | Kulkarni MA. et al., Malar J (2006) |
|  |  | *Anopheles culicifacies* | L1014F/S | Singh OP. et al., Malar J (2010) |
|  |  | *Anopheles gambiae* | L1014F/S | Martinez-Torres D. et al., Insect Mol Biol (1998) |
|  |  | *Anopheles peditaeniatus* | L1014S | Chaumeau V. et al., Parasit Vectors (2017) |
|  |  | *Anopheles sinensis* | L1014W | Tan. et al., (2012) |
|  |  | *Anopheles sinensis* | L1014S/F/C | Chang X. et al., Parasit Vectors (2016) |
|  |  | *Anopheles stephensi* | L1014F/S | Singh OP. et al., Malar J (2012) |
|  |  | *Culex pipiens* | L1014S/F/C | Wang ZM. et al., Med Vet Entomol (2012) |
|  |  | *Culex quinquefasciatus* | L1014F | Xu Q. et al., Biochem Biophys Res Commun (2006) |
|  |  | *Haematobia irritans* | L1014F | Guerrero FD. et al., Insect Biochem Mol Biol (1997) |
|  |  | *Musca domestica* | L1014F/H | Williamson MS.et al.,Mol Gen Genet(1996) |
|  |  | *Phlebotomus argentipes* | L1014F/S | Gomes B. et al., PLoS Negl Trop Dis (2017) |
|  |  | *Stomoxys calcitrans* | L1014H | Olafson PU. et al., J Econ Entomol (2011) |
|  |  | *Tuta absoluta* | L1014F | Haddi K. et al., Insect Biochem Mol Biol (2012) |
|  |  | *Cimex hemipterus* | L1014F | Dang K. et al., Pest Manag Sci (2015) |
|  |  | *Myzus persicae* | L1014F | Cassanelli. et al., (2005) |
|  |  | *Sitobion avenae* | L1014F | Foster SP. et al., Pest Manag Sci (2014) |
|  |  | *Triatoma infestans* | L1014F | Fabro J. et al., Infect Genet Evol (2012) |

**Table S2.** Continued.

| **Gene** | **Position^a^** | **Insect species** | **Mutation** | **References** |
| --- | --- | --- | --- | --- |
| ***VGSC*** | 1014 | *Cydia pomonella* | L1014F | Franck P. et al., PLoS One (2012) |
|  |  | *Helicoverpa zea* | L1029H | Hopkins BW. et al., Insect Biochem Mol Biol (2010) |
|  |  | *Heliothis virescens* | L1014H | Park Y. et al., Insect Biochem Mol Biol (1997) |
|  |  | *Plutella xylostella* | L1014F | Schuler. et al., (1998) |
|  |  | *Spodoptera frugiperda* | L1014F | Carvalho RA. et al., PLoS One (2013) |
|  |  | *Thrips tabaci* | L1014F | Toda S. et al., J Econ Entomol (2009) |
|  | 1016 | *Aedes aegypti* | V1016G/I | Brengues C. et al., Med Vet Entomol (2003) |
|  | 1020 | *Plutella xylostella* | F1020S | Endersby NM. et al., Bull Entomol Res (2011) |
|  | 1101 | *Plutella xylostella* | A1101T | Sonoda S. et al., Pest Manag Sci (2010) |
|  | 1410 | *Drosophila melanogaster* | A1410V | Pittendrigh B. et al., Mol Gen Genet (1997) |
|  | 1494 | *Drosophila melanogaster* | A1494V | Pittendrigh B. et al., Mol Gen Genet (1997) |
|  | 1524 | *Drosophila melanogaster* | M1524I | Pittendrigh B. et al., Mol Gen Genet (1997) |
|  | 1532 | *Aedes albopictus* | I1532T | Xu J. et al., PLoS Negl Trop Dis (2016) |
|  | 1534 | *Aedes aegypti* | F1534C | Harris AF. et al., Am J Trop Med Hyg (2010) |
|  |  | *Aedes albopictus* | F1534S | Xu J. et al., PLoS Negl Trop Dis (2016) |
|  | 1549 | *Helicoverpa armigera* | D1549V | Head DJ. et al., Insect Mol Biol (1998) |
|  | 1553 | *Helicoverpa armigera* | E1553G | Head DJ. et al., Insect Mol Biol (1998) |
|  | 1594 | *Culex quinquefasciatus* | W1594R | Li T. et al., Sci Rep (2012) |
|  | 1763 | *Aedes aegypti* | D1763Y | Chang C. et al., Insect Biochem Mol Biol (2009) |
|  | 1879 | *Plutella xylostella* | P1879S | Sonoda S. et al., Pest Manag Sci (2010) |
|  | 1999 | *Blattella germanica* | P1999L | Liu Z. et al., Insect Biochem Mol Biol (2002) |

Position^a^ : numbering according to *VGSC* of *Musca domestica*.
