# Supplementary material for: FastD: Fast detection of insecticide target‐site mutations and overexpressed detoxification genes in insect populations from RNA‐Seq data
Source: Ecol Evol. 2020 Nov 21;10(24):14346–58. doi: 10.1002/ece3.7037 (PMC7771117; doi:10.1002/ece3.7037)
Supplement: Supplementary file 3 — Table S3 [file ECE3-10-14346-s003.docx]

Table S3 Resistance-associated mutations in *RyR* of insects

| **Gene** | **Position^a^** | **Insect species** | **Mutation** | **References** |
| --- | --- | --- | --- | --- |
| ***RyR*** | 1338 | *Plutella xylostella* | E1338D | Guo L. et al., Sci Rep (2014) |
|  | 4946 | *Plutella xylostella* | Q4594L | Guo L. et al., Sci Rep (2014) |
|  | 4790 | *Plutella xylostella* | I4790M | Guo L. et al., Sci Rep (2014) |
|  |  | *Tuta absoluta* | I4746T | Roditakis E. et al., Insect Biochem Mol Biol (2017) |
|  | 4946 | *Plutella xylostella* | G4946E | Troczka B. et al., Insect Biochem Mol Biol (2012) Guo L. et al., Pest Manag Sci (2014) |
|  |  | *Tuta absoluta* | G4903V | Roditakis E. et al., Insect Biochem Mol Biol (2017) |
|  |  | *Chilo suppressalis* | G4910E | Yao R. et al., Pest Manag Sci (2017) |

Position^a^ : numbering according to *RyR* of *Plutella xylostella*.
