# Supplementary material for: FastD: Fast detection of insecticide target‐site mutations and overexpressed detoxification genes in insect populations from RNA‐Seq data
Source: Ecol Evol. 2020 Nov 21;10(24):14346–58. doi: 10.1002/ece3.7037 (PMC7771117; doi:10.1002/ece3.7037)
Supplement: Supplementary file 4 — Table S4 [file ECE3-10-14346-s004.docx]

Table S4 Resistance-associated mutations in *nAChR* of insects

| **Gene** | **Position** | **Insect species** | **Mutation** | **References** |
| --- | --- | --- | --- | --- |
| ***nAChR*** | α1-151^a^ | *Nilaparvata lugens* | α1-Y151S | Liu Z. et al., Proc Natl Acad Sci U S A (2005) |
|  | α3-151^b^ | *Nilaparvata lugens* | α3-Y151S | Liu Z. et al., Proc Natl Acad Sci U S A (2005) |
|  | α6-275^c^ | *Drosophila melanogaster* | α6-G275E | Perry T. et al., Insect Biochem Mol Biol (2007) |
|  |  | *Frankliniella occidentalis* | α6-G275E | Puinean AM.et al., J Neurochem (2013) |
|  |  | *Thrips palmi* | α6-G275E | Bao WX. et al., Pestic Biochem Physiol (2014) |
|  |  | *Tuta absoluta* | α6-G275E | Silva WM. et al., Pestic Biochem Physiol (2016) |
|  | β1-81^d^ | *Aphis gossypii* | β1-R81T | Zhang J. et al., Pestic Biochem Physiol (2015) |
|  |  | *Myzus persicae* | β1-R81T | Bass C. et al., BMC Neurosci (2011) |

| a: numbering according to *nAChR* α1 subunit of *Nilaparvata lugens* |
| --- |
| b: numbering according to *nAChR* α3 subunit of *Nilaparvata lugens* |
| c: numbering according to *nAChR* α6 subunit of *Frankliniella occidentalis* |
| d: numbering according to *nAChR* β1 subunit of *Aphis gossypii* |
